# Poliovirus Receptor as a Potential Target in Gastric Signet-Ring Cell Carcinoma for Antibody-Drug Conjugate Development

**DOI:** 10.3390/cancers18020270

**Published:** 2026-01-15

**Authors:** Yinxia Zhao, Hanfei Xie, Xuefei Tian, Li Yuan, Can Hu, Yujie Dai, Shengjie Zhang, Peng Guo, Xiangdong Cheng

**Affiliations:** 1 Faculty of Medicine, School of Life Sciences, Tianjin University, Tianjin 300072, China; 2022226029@tju.edu.cn; 2 Department of Gastric Surgery, Zhejiang Cancer Hospital, Hangzhou Institute of Medicine (HIM), Chinese Academy of Sciences, Hangzhou 310022, China; yuanli2768@zjcc.org.cn (L.Y.); hucan@zjcc.org.cn (C.H.); zhangsj@zjcc.org.cn (S.Z.); 3 Zhejiang Provincial Research Center for Upper Gastrointestinal Tract Cancer, Zhejiang Cancer Hospital, Hangzhou 310022, China; 4 Zhejiang Key Lab of Prevention, Diagnosis and Therapy of Upper Gastrointestinal Cancer, Zhejiang Cancer Hospital, Hangzhou 310022, China; 5 Clinical and Translational Research Center, Hangzhou Institute of Medicine (HIM), Chinese Academy of Sciences, Hangzhou 310018, China; xhf18255842225@163.com (H.X.); xftian24@m.fudan.edu.cn (X.T.); yc48627@um.edu.mo (Y.D.); 6 Department of Gynecologic Oncology, Zhejiang Cancer Hospital, Postgraduate Training Base Alliance of Wenzhou Medical University, Hangzhou 310022, China; 7 School of Molecular Medicine, Hangzhou Institute for Advanced Study, University of Chinese Academy of Sciences (UCAS), Hangzhou 310022, China; 8 Department of Biological Medicines and Shanghai Engineering Research Center of Immunotherapeutics, Fudan University, Shanghai 201203, China; 9 MOE Frontier Science Centre for Precision Oncology, Faculty of Health Sciences, University of Macau, Macau SAR 999078, China

**Keywords:** PVR, GSRCC, ADC, targeted therapy, proteomics analysis

## Abstract

Background: Gastric signet-ring cell carcinoma (GSRCC) is a distinct subtype of gastric cancer characterized by unique biological features, leading to low rates of early diagnosis, poor prognosis, and limited response to chemotherapy and immunotherapy. Effective targeted therapies for GSRCC remain scarce. Given these treatment challenges and the potential efficacy of antibody-drug conjugates (ADCs) in clinical settings, this study focuses on identifying novel ADCs with significant potential to improve the treatment outcomes of GSRCC. Methods: We conducted a comprehensive bioinformatics analysis of GSRCC using multi-omics data (including transcriptomics and proteomics) and identified the poliovirus receptor (PVR) as a potential therapeutic target for GSRCC. We selected deruxtecan (DXd) as an effective carrier for developing an ADC targeting GSRCC. The synthesized PVR monoclonal antibody-DXd complex (PVR-DXd) has a drug-to-antibody ratio (DAR) of 4. Results: PVR-DXd demonstrated potent antitumor activity in a human GSRCC xenograft model, effectively eliminating tumors while sparing normal tissue, highlighting its potential as a novel and impactful targeted therapy for this aggressive subtype of gastric signet ring cell carcinoma. Conclusions: This preliminary study supports the further development of PVR-DXd as a candidate therapy for advanced GSRCC.

## Simple Summary

Gastric signet ring cell carcinoma is a rare and aggressive form of stomach cancer that responds poorly to existing treatments. Many patients are diagnosed at an advanced stage and have limited therapeutic options. In this study, we analyzed cancer tissue at multiple molecular levels and identified a surface protein called the poliovirus receptor (PVR) as a promising target for new therapy. We then developed an antibody-drug conjugate (ADC) that links an antibody against PVR to a potent chemodrug, allowing the ADC to be delivered selectively into cancer cells. In animal models that closely mimic human disease, this targeted therapy strongly suppressed tumor growth while sparing normal tissues. Our findings suggest that this strategy may offer a more effective and safer treatment option for patients with this difficult-to-treat stomach cancer.

## 1. Introduction

Gastric signet-ring cell carcinoma (GSRCC) is a distinct and aggressive subtype of gastric cancer associated with significantly poorer survival outcomes. According to the World Health Organization classification, signet-ring cell carcinoma is defined as a poorly cohesive carcinoma primarily composed of tumor cells characterized by abundant cytoplasmic mucin and eccentrically placed crescent-shaped nuclei [1]. To date, GSRCC has become an increasingly prevalent subtype of gastric cancer, now accounting for approximately 16% of gastric cancer [2]. This rising prevalence underscores a growing clinical importance of GSRCC and the need for effective therapeutic strategies [3,4]. GSRCC exhibits an aggressive clinical course due to its highly invasive and metastatic nature, leading to a median survival of less than one year in advanced-stage patients [1,5]. 5-year overall survival (OS) rate for patients with advanced-stage GSRCC is less than 20% in comparison with approximately 30–50% for intestinal-type gastric cancer [6]. Early detection remains challenging due to the absence of identifiable precancerous lesions and nonspecific symptoms. Moreover, it is challenging to accurately quantify the proportion of signet-ring cells in biopsy specimens. Over 80% of GSRCC cases are diagnosed at advanced stages (III or IV), further limiting curative treatment options [7]. GSRCC differs pathologically from gastric adenocarcinoma, often presenting in younger female patients and more commonly occurring in the middle and lower thirds of the stomach [8]. Current treatment strategies for GSRCC remain controversial, as most studies do not specifically stratify GSRCC from other gastric cancer subtypes. Targeted therapies for diffuse gastric cancer, including anti-HER2 and anti-VEGFR2 agents, have demonstrated limited benefit, with incremental improvements in median OS of less than 3 months in advanced cases [9,10]. These outcomes underscore the urgent need for novel molecular targets and therapeutic strategies specifically tailored to GSRCC.

Antibody-drug conjugates (ADCs) represent a promising approach to addressing these challenges. ADCs combine the tumor specificity of monoclonal antibodies with the potent cytotoxicity of small-molecule drugs, allowing selective delivery to primary tumors and metastatic lesions while sparing normal tissues. Microsatellite Instability (MSI) and HER2 status can serve as biomarkers to guide preoperative treatment strategies for advanced gastric cancer [11]. Trastuzumab deruxtecan, an ADC targeting HER2, has been FDA-approved for advanced HER2-positive gastric cancer, demonstrating an objective response rate (ORR) of over 40% and a median progression-free survival of 5.6 months [12]. Other ADC targets under investigation for gastric cancer include HER3, Guanylate Cyclase C (GCC), Trop-2, and Claudin 18.2 [13]. However, to date, no ADCs have yet been designed or evaluated specifically for GSRCC. Thus, here we identified poliovirus receptor (PVR, also known as CD155) as a novel and promising target for GSRCC through a comprehensive multi-omics analysis. PVR is a transmembrane glycoprotein in the nectin-like family, with physiological roles in cell adhesion, migration, and immune regulation, PVR is frequently upregulated in cancer, where it is strongly associated with tumor progression, metastasis, and immune evasion [14,15,16,17]. Quantitative studies have shown that PVR expression correlates with advanced stages of disease, with high expression levels linked to a 30% reduction in OS in multiple cancer types, including colorectal, pancreatic, and gastric cancers [16,18,19]. In the tumor microenvironment (TME), PVR interacts with immune checkpoint molecules such as TIGIT, inhibiting the activity of natural killer (NK) cells and T cells, thereby suppressing anti-tumor immunity [14,15,16,17,20]. The dual role of PVR in enhancing tumor cell survival and mediating immune evasion makes it a compelling target for therapeutic interventions. Its negligible expression in normal tissues further supports its potential for selective targeting. Herein, we leveraged these insights to explore the potential of PVR as a therapeutic target for GSRCC, providing robust preclinical evidence to support the development of PVR-targeted ADCs aimed at improving clinical outcomes for patients with this aggressive cancer subtype.

## 2. Materials and Method

### 2.1. Cell Lines and Cell Culture

Human GSRCC cell lines, including NUGC4, KATO3 and human gastric adenocarcinoma cell lines MKN45 were purchased from Meisen CTCC (Hangzhou, China), NUGC4, MKN45, KATO3 cells were cultured in Roswell Park Memorial Institute (RPMI)-1640 containing 10% fetal bovine serum and 1% penicillin/streptomycin, and the cells were cultured under a humidified atmosphere of 5% CO_2_ at 37 °C.

### 2.2. Bioinformatic Analysis

Proteins suitable for ADC were identified through the proteomic analysis of cancer tissues and adjacent tissues of 125 patients with GSRCC in Zhejiang Cancer Hospital. Candidate proteins were further validated through The Human protein Atlas database (https://www.proteinatlas.org (accessed on 2 November 2025)) data analysis and microbiogenic analysis (SRplot: A free online platform for data visualization and graphing (https://journals.plos.org/plosone/article?id=10.1371/journal.pone.0294236, accessed on 26 October 2025)). The PVR expression in TCGA cohort was assessed in Gene expression Profiling Interactive Analysis 2 (GEPIA2) (GEPIA (Gene Expression Profiling Interactive Analysis (http://gepia.cancer-pku.cn/, accessed on 26 October 2025))) [21]. Target Screening and Identification: Specific criteria for screening ideal ADC targets based on multi-omics (transcriptome and proteome) data: High expression: Expression fold change in GSRCC tissue > 2 compared to adjacent non-cancerous tissue, with expression levels in the top 20%; Membrane localization: Confirmed as a membrane protein via immunohistochemistry images from The Human Protein Atlas and our IHC results; Low expression in normal tissues: Low expression levels in key normal organs (heart, liver, kidney, etc.); Internalization capacity: Demonstrated good internalization properties through our prior internalization experiments.

### 2.3. Flow Cytometric (FCM)

2 × 10^6^ cells were collected and divided into two groups. The cells were resuspended in 1 mL of phosphate buffered saline (PBS) and centrifuged at 1200 rpm for 5 min. Under ice bath conditions, the cells were blocked with PBS containing 1% bovine serum albumin (BSA) for 30 min [22]. After blocking, the cell suspensions were centrifuged again at 1200 rpm for 5 min. The cell pellets were resuspended in 800 µL of PBS containing 1% BSA and further divided into two groups: the IgG group and the PVR group. 2 µL of PE-conjugated Mouse IgG (Cat#400114, BioLegend, San Diego, CA, USA) or PE-conjugated anti-human CD155 (Cat#337610, BioLegend, San Diego, CA, USA) was added to 200 µL of the cell suspension. The cells were incubated for 45 min at room temperature on a shaking platform set at 1200 rpm, protected from light. Following incubation, the cells were washed once with 1 mL of PBS to remove unbound antibodies. The cells were then resuspended in 0.5 mL of PBS and analyzed using a CytoFLEX LX Flow Cytometer (Beckman Coulter, San Diego, CA, USA).

### 2.4. Immunofluorescent (IF) Staining

1 × 10^6^ GSRCC cells were blocked with PBS containing 1% BSA for 15 min under ice bath conditions and then divided into three groups: PBS group, IgG group, and PVR group. The PBS group was incubated in 1 mL PBS for 1 h, the IgG group was incubated with 2 µL FITC-conjugated Mouse IgG (Cat#M212308S, Abmart, Shanghai, China) and 1 mL PBS for 1 h, and the PVR group was incubated with 2 µL FITC-conjugated anti-human CD155 antibody (Cat#81254, CST, Shanghai, China) and 1 mL PBS for 1 h. Following incubation, the cells were washed once with PBS to remove unbound antibodies and stained with Hoechst staining solution (Biosharp, Hefei, China) at 37 °C for 20–30 min. After staining, the cells were washed twice with PBS to remove excess dye and observed at 488 nm using a Nikon A1 HD25 laser confocal microscope (Nikon, Minato Ward, Japan).

### 2.5. Cellular Internalization of PVR Monoclonal Antibody

Quantitative evaluation using flow cytometry (FCM) was performed on 1 × 10^7^ GRSCC cells, which were divided into four groups corresponding to different time points (0 min, 60 min, 120 min, and 240 min). The cells were resuspended in PBS and centrifuged at 1200 rpm for 5 min. Each group was incubated with Purified anti-human CD155 (Cat#337610, BioLegend, San Diego, CA, USA) on ice for 30 min. Following incubation, the cells were washed once with 1 mL PBS to remove unbound primary antibodies. For the 0 min group, 1 mL PBS and 2 µL secondary antibody PE anti-mouse IgG (Cat#406608, BioLegend, San Diego, CA, USA) were immediately added, and the cells were incubated on ice for 30 min. Afterward, the cells were washed once with PBS to remove unbound secondary antibodies, fixed with 4% paraformaldehyde (Solarbio, Beijing, China) for 10 min, washed again with PBS, and resuspended in 500 µL PBS. The remaining three groups underwent endocytosis at 37 °C in 1 mL PBS for the respective time intervals (60 min, 120 min, and 240 min) after the addition of the secondary antibody, following the same procedure as the 0 min group. Flow cytometry was then used to measure fluorescence intensity, and the percentage of endocytosis at time point t = 100 − MFI (mean fluorescence intensity) of t min samples × 100/MFI of 0 min samples.

Qualitative evaluation using immunofluorescence (IF) staining was conducted on 1 × 10^6^ GRSCC cells were stained with 1 mL PBS containing 2 µL PE anti-human PVR antibody for 30 min under ice bath conditions. Following staining, the cells were washed once with PBS and incubated for 0, 60, 120, and 240 min at 37 °C in PBS to allow endocytosis. After removing PBS, the cells were stained with 1 mL Hoechst solution at 37 °C for 10–15 min, washed twice with PBS, and fixed with 4% paraformaldehyde for 10 min. These prepared samples were then analyzed for endocytosis and localization.

### 2.6. Synthesis and Characterization of PVR-Targeted ADC

The antibody used for ADC preparation was produced by Baiying Bio (Shanghai, China). The amino acid sequence of the PVR antibody was obtained from the publicly available patent CN116284395A, and a human IgG4 subtype anti-PVR monoclonal antibody was synthesized accordingly. Plasmid constructs were designed to encode the heavy and light chains of the antibody. Both genes were codon-optimized, synthesized, and cloned into a dual-plasmid expression system (Plasmid 1: heavy chain; Plasmid 2: light chain) using appropriate restriction enzyme sites. CHO (Chinese hamster ovary) cells were selected as the expression host to ensure human-like glycosylation patterns. Transient transfection was performed using electroporation or chemical transfection. Monoclonal high-expressing cell lines were selected and expanded. The cells were cultured in suspension in serum-free medium at 37 °C, pH 7.2–7.4, with 5% CO_2_. Antibody expression levels were monitored by ELISA. The culture supernatant was harvested by centrifugation and filtered through a 0.22 μm membrane to remove cell debris. The antibody was purified using Protein A/G affinity chromatography. Bound antibodies were eluted using a low-pH buffer (pH 3.0), immediately neutralized, and subjected to buffer exchange into PBS (pH 7.2–7.4). 

The PVR-targeting ADC was subsequently prepared using a cleavable linker–payload, MC-GGFG-DXd (Cat# HHY-13631E, Shanghai, China). The reaction process involved breaking disulfide bonds of cysteine of PVR antibodies to form reactive thiol groups using reducing agent tris (2-carboxyethyl) phosphine (TCEP, Cat#51805-45-9, MCE, Shanghai, China) at a 1:4 molar ratio on a shaker set at 37 °C for 45 min [23]. Subsequently, MC-GGFG-DXd was added to the reaction mixture at a 1:10 molar ratio, followed by an overnight reaction under the same conditions. The reaction products were then subjected to dialysis using a dialysis bag (Cat#20536ES03, Yeasen biotechnology, Shanghai, China), with the buffer being replaced every two hours over a 24-h period. After dialysis, the product was verified through mass spectrometry using MALDI-TOF to confirm successful conjugation and characterization of the ADC [24,25]. The DAR calculation was based on MALDI-TOF mass spectrometry data, which showed that the measured molecular weight of the unconjugated antibody (PVR) is approximately 145 kDa, which aligns well with its theoretical mass (144.8 kDa). After conjugation with MC-GGFG-DXd, the major peak of the ADC appears at approximately 149 kDa, indicating a molecular weight increase of ~4 kDa. The payload molecule MC-GGFG-DXd has a known molecular weight of 1034.05 Da. Therefore, the DAR was calculated using the following formula:

DAR = (Molecular weight of ADC − Molecular weight of antibody)/Molecular weight of payload. Supporting data are provided in Supplementary Figures S1 and S2.

### 2.7. In Vitro Cytotoxicity

The in vitro cytotoxicity of PVR-DXd and controls (5-FU, PVR mAb, and IgG-DXd) was evaluated using the Cell Counting Kit-8 (CCK-8, Beyotime, Shanghai, China). Cells were seeded into 96-well plates at a density of 4 to 10 × 10^3^ cells per well, depending on the growth rate of the specific cell lines. Each well contained 90 µL of medium, and the plates were incubated for 24 h. Subsequently, 10 µL of drug solution was added to each well to achieve a series of diluted drug concentrations ranging from 10^−6^ to 10 µg/mL. The plates were then incubated for 72 h. Following the treatment period, 10 µL of CCK-8 solution was added to each well and incubated according to the manufacturer’s instructions. The absorbance was measured at 450 nm using a microplate reader. Half-maximal inhibitory concentration (IC_50_) values were calculated using GraphPad Prism 9.5.

### 2.8. Tumor-Specificity and Biodistribution of PVR Monoclonal Antibody

PVR monoclonal antibodies were labeled with Cy3 using an amine-NHS coupling reaction for subsequent in vitro studies. Briefly, 1 mg of PVR antibody was gently mixed with a 3 molar equivalent of Cy3 NHS ester (MedChemExpress, Monmouth Junction, NJ, USA). The reaction was carried out at 37 °C overnight in the dark. Following the reaction, Cy3-labeled PVR antibodies were purified usingultracentrifuge, and their concentrations were quantified using the BCA protein assay. All animal studies were conducted in compliance with protocols approved by the Institutional Animal Care and Use Committee of the Hangzhou Institute of Medicine, Chinese Academy of Sciences (Approval No. AP2024-10-0219). For in vivo biodistribution studies, 2 × 10^6^ NUGC4 cells were mixed with Matrigel and implanted via subcutaneous injection into right flank of nude mice (female, 4–6 weeks old). Once tumors were established, the NUGC4 tumor-bearing mice were randomly divided into two groups (N = 5 per group). Each group received an intravenous injection of either IgG-Cy3 or PVR-Cy3 at an equal dose of 5 mg/kg body weight. At 48 h post-injection, ex vivo bioluminescence imaging intensity in tumors and normal organs (heart, liver, spleen, lungs, and kidneys) was evaluated using the IVIS imaging system.

### 2.9. In Vivo Evaluation of the Antitumor Efficacy of PVR-DXd in Subcutaneous GSRCC Mouse Tumor Models

The human GSRCC cell line NUGC4 was used to establish subcutaneous tumors in female athymic nude mice (female, 4–6 weeks old). Each mouse was injected with 2 × 10^6^ cells into right flank. Once tumors reached an average volume of 100 mm^3^, mice were randomized into five groups (N = 6 per group): vehicle control (PBS), 5-FU, PVR monoclonal antibody (PVR mAb), IgG-DXd, and PVR-DXd. All treatments were administered intravenously at a dose of 5 mg/kg once weekly. Tumor size and body weight were measured and recorded twice weekly. Tumor volume was calculated using the formula Volume = ab^2^/2, where a is the longest diameter and b is the perpendicular shorter diameter. At the study endpoint, subcutaneous tumors were excised and weighed to determine tumor mass. Monitoring indicators including tumor volume, body weight, and tumor mass were assessed.

Paraffin sections were dewaxed to water: sequentially in environmental protection dewaxing agent, environmental protection dewaxing agent, environmental protection dewaxing agent (all purchased from Hubei Baios Biotechnology Co., Ltd., Wuhan, China.) for 10 min, respectively, and then by anhydrous ethanol, 95% ethanol, 85% ethanol, and 75% ethanol (Sinopharm Chemical Reagent Co., Ltd. 100092683, Shanghai, China) for 5 min each. Rinse with tap water for 1 min. Hematoxylin staining solution (Harris) (Hubei Baios Biotechnology Co., Ltd. BP0211) was stained for 4 min and washed with tap water for 2 min until no excess stain was removed from the sections. Alcohol differentiation with 0.8% hydrochloric acid for 2 s, rinsed with tap water, and lithium carbonate aqueous solution can also be used to return to the blue, and then washed with water for 2 min. Into the red dye solution (alcohol soluble) dye 20 s, without washing, directly into the 95% ethanol toning 5 s, into the anhydrous ethanol, anhydrous ethanol dehydration for 2 min. Environmental protection transparent agent transparent, sealing, microscopic examination. The results were interpreted, the nucleus was bluish-purple, the cytoplasm, interstitium and various fiber types were red in different degrees. Biochemical tests for liver and kidney function indicators were performed by Hubei Baios Biotechnology.

### 2.10. Statistical Analysis

The data were presented as the mean ± standard error of the mean (SEM). Statistical differences were evaluated using one-way or two-way ANOVA, as appropriate for the specific experiments, and analyzed with GraphPad Prism 9.5 software. Kaplan–Meier survival analysis was conducted using the log-rank test to determine statistical variance. A *p*-value of <0.05 was considered statistically significant.

## 3. Results

### 3.1. Identification of PVR as a Potential Target for GSRCC

To meet the safety and efficacy criteria for ADC targets, ADC targets should be highly expressed in tumor tissues and minimally or even not expressed in normal tissues. Our previous study reported the proteomic analysis of 125 GSRCC tumors and paired normal adjacent tissues (NATs).

**Figure 1:**
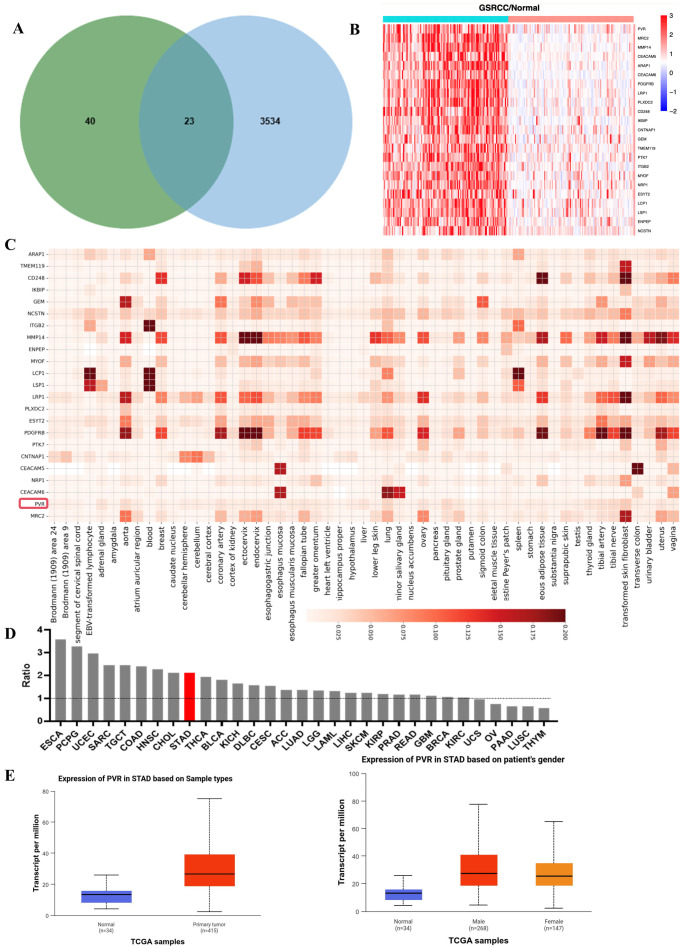
PVR was overexpressed in GSRCC. (**A**) Venn diagram of DEPs and membrane proteins from Cancer Surfaceome Atlas; (**B**) Heatmap of expression of DEPs between GSRCC tumor and paired NAT samples; (**C**) Heatmap of the normal tissue expression index of 23 candidate targets; (**D**) The PVR mRNA expression ratio between paracancerous tissues and cancers from GEPIA database; (**E**) PVR mRNA expression levels of human STAD tumor tissues and normal gastric tissues in TCGA samples acquired from UALCAN website. Error bars, SD. Unpaired *t*-test. Median, quartiles, minimum, and maximum values are represented by the central line, limits of box, and ends of lines of boxplots shown.

As illustrated in Figure 1A, the green circle on the left represents the 63 differentially expressed proteins (DEPs) identified through proteomic profiling of 125 GSRCC tumor tissues. The blue circle on the right denotes the 3534 membrane proteins cataloged in the Cancer Surfaceome Atlas (http://fcgportal.org/TCSA (accessed on 12 November 2025)) [18]. The intersection between the two circles highlights 23 DEPs that are localized to the cell membrane. In contrast, the remaining 40 DEPs, which do not overlap with the membrane protein dataset, are not membrane-localized. Since membrane localization is a critical requirement for ADC targeting, these non-membrane DEPs were excluded from subsequent analysis. Figure 1B illustrates these 23 differentially expressed membrane proteins expression in gastric indolent carcinoma tissues and paracancerous tissues from clinical patients. Next, we compared the expression levels of the top 23 DEPs localized to the cell membrane in 54 normal tissues in humans to assess their targeting and non-tumor toxicity potential, and the expression levels of PVRs in normal tissues, including the stomach, were consistently low (as shown in Figure 1C).

To assess tumor specificity, we calculated the ratio of tumor expression of PVRs to adjacent tissue expression in various cancer types using The Cancer Genome Atlas (TCGA) and GEPIA database. 

Due to the limited availability of transcriptomic datasets specific to GSRCC, we performed additional expression analysis using stomach adenocarcinoma (STAD) data from TCGA and GEPIA databases, which are widely used in gastric cancer research. While GSRCC represents a histopathological subtype of STAD, it is not independently annotated in these public databases. Therefore, STAD data were used as a proxy to assess broader expression patterns of PVR in gastric cancer.

From the GEPIA database, the tumor-to-normal expression ratio of PVR in gastric cancer exceeded two-fold (Figure 1D). Analysis of TCGA-STAD transcriptome data further confirmed that PVR expression was significantly elevated in tumor tissues (n = 415) compared to normal gastric tissues (n = 34). No significant differences in PVR expression were observed between male (n = 268) and female (n = 147) patients (Figure 1E). While the STAD dataset from TCGA provided a useful reference for validating our findings, we acknowledge that GSRCC comprises only a small subset (~10%) of this cohort and our conclusions specific to GSRCC were primarily derived from our dedicated cohort of 125 GSRCC tumors.

Collectively, these findings support PVR as a promising ADC target for GSRCC. Despite the use of STAD datasets due to the unavailability of GSRCC-specific transcriptomic profiles, the evidence points to PVR’s high tumor specificity and minimal expression in normal tissues, justifying further investigation in the GSRCC context.

### 3.2. Association Between PVR Expression and Immune Cell Infiltration in Gastric Adenocarcinoma

To explore the potential immunological role of PVR in GSRCC, we conducted a series of transcriptomic analyses including DEG analysis, pathway enrichment, and immune cell composition estimation using data from TCGA STAD cohort. Due to the absence of GSRCC-specific transcriptome data in public repositories, STAD samples were used as a proxy to infer immune-related associations in gastric cancer. However, we acknowledge that GSRCC represents a histologically and biologically distinct subtype of STAD, and findings from this cohort should be interpreted with appropriate caution.

**Figure 2:**
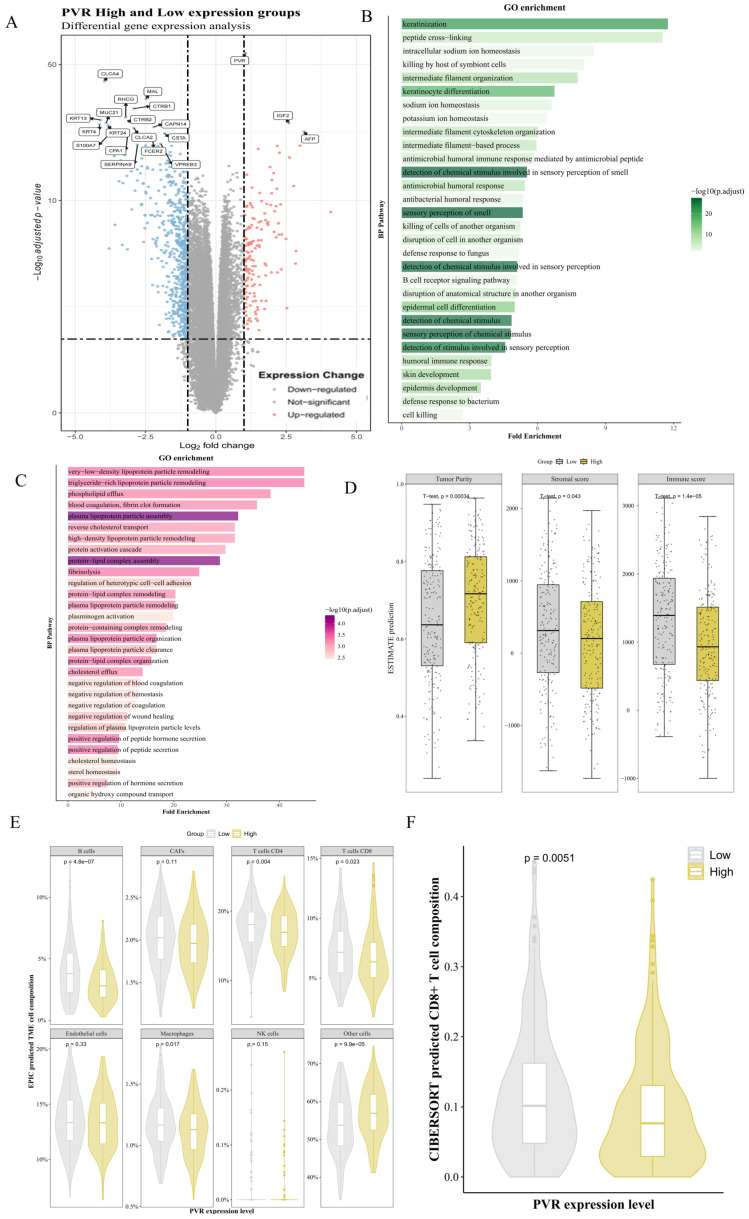
Immune Cell Landscape and PVR expression in GC patients. (**A**) Volcano map of high-PVR expression versus low-PVR expression group; (**B**) The top 30 pathways of Gene Ontology (GO) enrichment analysis; (**C**) The top 30 pathways of Reactome pathway analysis; (**D**) Tumor purity score, Stroma score and Immune score estimated by ESTIMATE between high-PVR expression group and low-PVR expression group; (**E**) Composition Estimated by EPIC Between High and Low PVR Expression Groups; (**F**) Proportion of CD8^+^ T cells estimated by CIBERSORT between high-PVR expression group and low-PVR expression group.

Samples were divided into PVR high- and low-expression groups based on the median PVR expression level. The resulting volcano plot (Figure 2A) shows DEGs between the two groups, filtered using a threshold of logFC > 1 and *p* < 0.05. Gene Ontology Biological Process (GO BP) enrichment analysis of these DEGs (Figure 2B,C) revealed that upregulated pathways were enriched for lipid metabolism and lipoprotein remodeling, while downregulated pathways were predominantly associated with immune functions, especially humoral immune responses. These results suggest a potential link between high PVR expression and reduced immune activity in the STAD cohort [26,27].

To further assess the TME, immune and stromal scores were estimated using the ESTIMATE algorithm. High PVR expression was associated with significantly lower immune scores and higher tumor purity (Figure 2D). These results were further supported by immune cell deconvolution using the EPIC and CIBERSORT algorithms, both of which indicated reduced CD8^+^ T cell infiltration in tumors with high PVR expression (Figure 2E,F, *p* < 0.05).

While these analyses do not establish a mechanistic role for PVR in immune modulation, and were conducted in a mixed STAD population, they raise the possibility that elevated PVR expression may be associated with a less immunologically active tumor microenvironment. Further validation in GSRCC-specific cohorts and functional studies will be necessary to determine whether similar trends hold true and whether PVR actively contributes to immune evasion in this subtype.

### 3.3. Evaluation of PVR Expression and Internalization in GSRCC In Vitro and In Vivo

**Figure 3:**
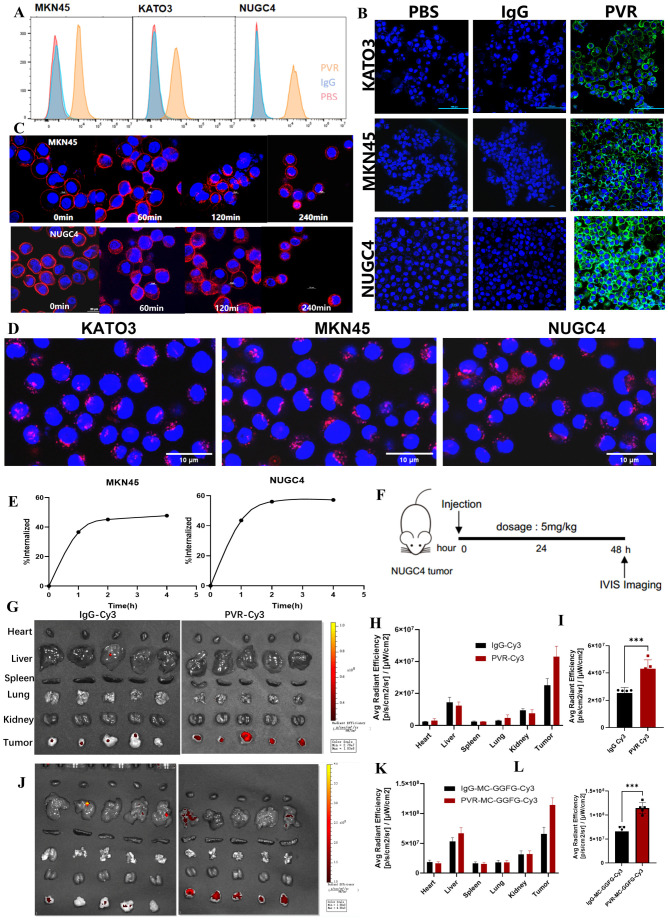
Identification of PVR as a GSRCC target. (**A**) Evaluation of PVR expression on human GSRCC cells indicated by FACS; (**B**) IF staining of PVR in MKN45, KATO3 and NUGC4 cells. Scale bar is 100 μm; (**C**) Internalization of PVR Ab by MKN45 and NUGC4 were detected by IF staining within 4 h. Scale bar is 20 μm; (**D**) Cell internalization assay using pHrodo Red-labeled PVR antibodies; (**E**) Line charts showing the internalization of PVR Ab by MKN45 and NUGC4 acquired by FACS; (**F**) Schematic diagram of biodistribution assay strategy; (**G**) Tumor-bearing mice were dissected to observe detailed biodistribution profile in organs containing heart, liver, spleen, lung, kidney and tumor; (**H**) Quantified the biodistribution of IgG-Cy3, PVR-Cy3, in normal organs (heart, liver, spleen, lung, kidney) and tumor. Data are shown as mean ± s.e.m. NS not significant; (**I**) Average fluorescent intensity of IgG-Cy3 and PVR-Cy3 in tumors, Unpaired *t*-test, *** *p* < 0.001; (**J**) Ex vivo biodistribution of PVR-MC-GGFG-Cy3 in each organ of heart, liver, spleen, lung, kidney and tumor; (**K**) Quantified the biodistribution of IgG-MC-GGFG-Cy3, PVR-MC-GGFG-Cy3 in normal organs (heart, liver, spleen, lung, kidney) and tumors. Data are expressed as mean ± s.e.m., NS not significant; (**L**) Mean fluorescence intensity of IgG-MC-GGFG-Cy3 and PVR-MC-GGFG-Cy3 in tumors, Unpaired *t*-test, *** *p* < 0.001.

We determined the membrane expression of PVR in two established human GSRCC cell lines (KATO3, NUGC4) and one human gastric adenocarcinoma cell line (MKN45) using flow cytometry (FCM). As shown in Figure 3A, NUGC4 cells exhibited the highest level of PVR expression among the tested cell lines, followed by MKN45 and KATO3. To corroborate these findings, we performed immunofluorescence (IF) staining, which demonstrated consistent results with FCM, showing that PVR was predominantly localized on the plasma membrane of the GSRCC cell lines tested (Figure 3B). This prominent membrane localization of PVR highlights its accessibility for PVR-targeted therapeutics. Moreover, the binding affinity of PVR antibodies were determined as 5.48 × 10^−10^ M by surface plasmon resonance (SPR, Figure S3). Consistent with this tumor-selective expression pattern, immunohistochemistry data from the Human Protein Atlas database (www.proteinatlas.org (accessed on 22 November 2025)) show that PVR protein is undetectable in normal lung alveolar epithelium, liver, brain, and colon, with only weak staining in a small fraction of bone-marrow cells and lung macrophages, while immune-cell RNA profiles indicate enrichment mainly in myeloid subsets rather than T or NK cells; together with the use of an IgG4 Fc-silenced backbone in our ADC design, which minimizes Fcγ receptor–mediated ADCC/CDC and macrophage engagement, these findings support a restricted normal-tissue distribution and a low risk of on-target pulmonary or hematologic toxicity.

Although PVR was widely distributed on cancer cell membranes, binding affinity alone does not guarantee effective internalization, a critical factor for ADC efficacy. To address this, we quantitatively and qualitatively assessed the internalization activity of PVR antibodies in GSRCC cells through FCM and IF analysis. As shown in the IF staining images, we observed PVR monoclonal antibody (mAb) staining initially localized on the cell surface, with significant intracellular fluorescence signals emerging over time. After 4 h, the fluorescence intensity on the cell surface decreased by approximately 50% in MKN45 cells and by 60% in NUGC4 cells, indicating efficient internalization of PVR mAb (Figure 3C,E). Additionally, a pHrodo Red-based internalization assay was also performed to validated the effective endocytosis of PVR antibodies in human GSRCC cells (Figure 3D).

Next, we validated the GSRCC tumor specificity of PVR mAb in vivo. We performed GSRCC tumor and organ biodistribution studies of PVR mAb using a mouse xenograft model carrying NUGC4 tumors (Figure 3E). PVR mAb and non-targeting IgG were labelled with Cy3 fluorophores (PVR-Cy3 and IgG-Cy3) and systemically exposed by intravenous injection. IVIS bioluminescence imaging at 48 h post-injection showed a significant two-fold increase in PVR-Cy3 accumulation at the GSRCC tumor site compared to the IgG control. Autopsy and analysis of major organs (heart, liver, spleen, lungs and kidneys) confirmed that PVR-Cy3 preferentially accumulates in tumors, whereas much lower fluorescence signals were observed in normal organs (Figure 3F–H). We then complemented the sulfhydryl coupling experiments of the PVR antibody with MC-GGFG-Cy3 by using the same sulfhydryl coupling as the DXd coupling approach, and repeated the experimental procedure as described above, and IVIS bioluminescence imaging showed that the accumulation of PVR-MC-GGFG-Cy3 at the GSRCC tumor site was still significantly increased by approximately two-fold compared to the IgG control (Figure 3I). Ex vivo imaging of major organs (heart, liver, spleen, lungs, and kidneys) confirmed that PVR-MC-GGFG-Cy3 predominantly accumulates in tumors, whereas the fluorescence signal was substantially lower in normal organs (Figure 3J–L). This tumor-selective distribution highlights the potential of PVR mAb in GSRCC-targeted therapy. These findings suggest that PVR, with its high membrane expression, potent internalization activity and tumor-selective biodistribution, is a promising cell membrane protein target for the design of ADCs against GSRCC.

### 3.4. Synthesis, Characterization, and Therapeutic Potential of PVR-DXd for GSRCC

**Figure 4:**
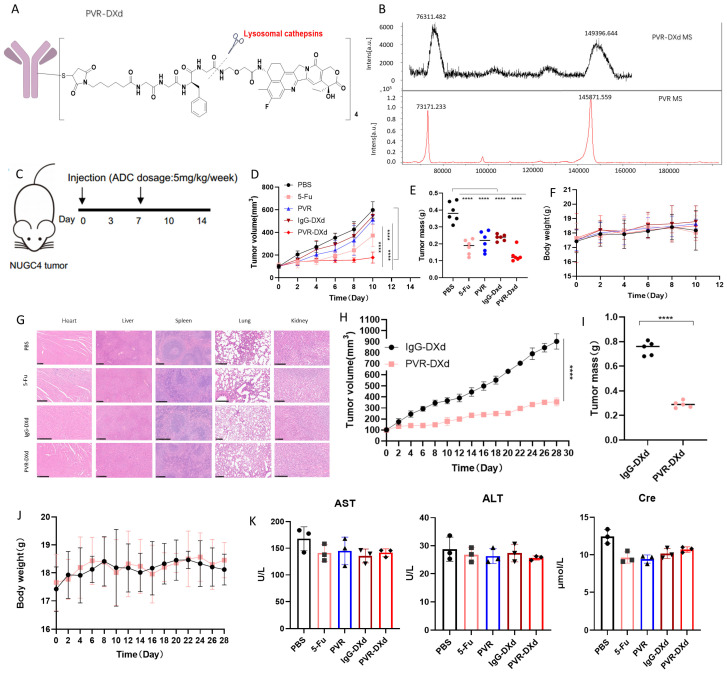
Synthesis of PVR-DXd and in vitro efficacy of PVR-DXd. (**A**) Schematic diagram of the structure of an PVR-DXd; (**B**) PVR and PVR-DXd molecular weight beating mass spectra were used to determine DAR values. (**C**) Timeline of treatment protocol in a GSRCC cell line (NUGC4) subcutaneous tumor model; (**D**) Tumor progression in the GSRCC tumor (NUGC4) subcutaneous tumor model treated with PBS, 5-Fu, PVR Ab, IgG-DXd, and PVR-DXd, respectively, monitored by tumor volume measurement (N = 6 per group). Error bars, SEM; (**E**) Quantified the tumor mass at end point (day 12); (**F**) Body weight trends in tumor-bearing mice during treatment (12 days). (**G**) HE staining of major organs, including heart, liver, spleen, lung and kidney in PBS, 5-Fu, IgG-DXd and PVR-DXd groups. Scale bar 250 μm; (**H**) Tumor progression in the GSRCC tumor (NUGC4) subcutaneous tumor model treated with IgG-DXd and PVR-DXd, respectively, monitored by tumor volume measurements (5 mice per group). Error bars, SEM; (**I**) Quantification of tumor mass at the endpoint (day 28), Unpaired *t*-test, **** *p* < 0.0001; (**J**) Trend in body weight of tumor-bearing mice during treatment (day 28). (**K**) Blood chemistry parameters (liver function: AST and ALT; kidney function: Cre measured in PBS, 5-Fu, PVR, IgG-DXd and PVR-DXd groups).

To exploit the acceptability of PVR as an ADC target, we synthesized PVR-GGFG-DXd doped with a potent cytotoxic payload, DXd (PVR-DXd), via a cysteine-maleimide linkage, as shown in Figure 4A, the DAR of PVR-DXd was determined to be approximately 4 using MALDI-TOF mass spectrometry. The MALDI-TOF data showed that the measured molecular weight of the unconjugated PVR antibody was 145 kDa, with a single, sharp peak as expected(Figure 4B). After conjugation with MC-GGFG-DXd, the molecular weight of the main peak shifted to 149 kDa, indicating an increase of approximately 4 kDa. Given that the molecular weight of the MC-GGFG-DXd payload is 1034.05 Da, the calculated DAR is approximately 4.

We evaluated the efficacy of PVR-DXd in vivo using a nude mouse NUGC4 xenograft model as shown in Figure 4C. NUGC4 tumor-bearing mice were randomized into groups receiving PBS, 5-FU, PVR-mAb, IgG-DXd, or a once-weekly intravenous injection of a 5 mg/kg dose of PVR-DXd. Throughout the study period, rapid tumor progression was observed in the PBS group, whereas significant tumor regression was observed in the PVR-DXd treatment group. Tumor volume was significantly smaller in the PVR-DXd group compared to the PBS and 5-FU groups. Antagonizing PVRs with the PVR antibody alone may exert moderate antitumor effects, whereas the IgG-DXd control also exhibited similar impact on tumor volume (Figure 4D). Quantitative measurements of tumor weight confirmed these findings, with mean tumor weights of 0.39 g (PBS), 0.18 g (5-FU), 0.21 g (PVR-mAb), 0.24 g (IgG-DXd) and 0.13 g (PVR-DXd), respectively (Figure 4E). Importantly, no significant weight loss was observed in any of the treatment groups, suggesting that no significant toxicity was observed during the study period (Figure 4F).

PVR-DXd-related toxicity in major organs (heart, lungs, liver, spleen and kidney) was assessed by histological analysis and blood biochemistry. Histopathological analysis of major organs (heart, liver, spleen, lungs and kidney) using H&E staining revealed no significant necrotic or degenerative changes in any of the treatment groups, which further confirmed the biosafety of PVR-DXd (Figure 4G). Tumor progression was monitored over 28 days in the subcutaneous GSRCC (NUGC4) tumor model following treatment with either IgG-DXd or PVR-DXd. The PVR-DXd treatment group exhibited sustained tumor growth inhibition, with approximately a 39% reduction in tumor volume compared to the control group (* *p* < 0.01) by day 28. No signs of treatment resistance or toxicity rebound were observed throughout the study period (Figure 4H–J). Statistical analysis of long-term efficacy confirmed that the tumor-suppressive effect of ADC remained stable over time. In addition, the survival rate of mice in the treatment group was significantly higher than that of the control group in the long-term observation (* *p* < 0.05 **), supporting the continued effectiveness of this ADC. Serum biochemical analyses showed no significant differences in liver function indices (ALP and AST) or renal function indices (Cre) between the groups, indicating minimal off-target toxicity in vivo (Figure 4K). In conclusion, our results suggest that PVR-DXd is a potent ADC with strong in vivo antitumor activity against GSRCC. Compared with the control group, the PVR-DXd group showed significant tumor shrinkage and minimal systemic toxicity. These findings highlight the potential of PVR-DXd as an effective and safe therapeutic option for GSRCC.

## 4. Discussion

In this study, we conducted a comprehensive multi-omics analysis of GSRCC to identify the membrane target PVR and synthesized the corresponding ADC drug, PVR-DXd. Our findings demonstrate that PVR-DXd exhibits significant anti-tumor efficacy with limited toxicity in a GSRCC tumor model. This study provides a paradigm for the identification of specific membrane targets and the synthesis of ADCs with tailored payloads for particular cancers, advancing the development of targeted cancer therapies.

Patients with GSRCC typically face a poor prognosis due to the aggressive nature of the disease, diagnostic challenges, and limited therapeutic options. GSRCC is characterized by a diffuse infiltration pattern, often leading to linitis plastica [28], which complicates complete surgical resection and results in residual disease. Its invasive growth and high metastatic potential, particularly through lymphatic and peritoneal spread, contribute to rapid disease progression. Moreover, GSRCC exhibits resistance to apoptosis and chemoresistance to conventional treatments such as 5-fluorouracil and platinum-based therapies, partly due to its low proliferative index and intrinsic molecular mechanisms. These factors reduce the effectiveness of standard chemotherapy.

The development of GSRCC-targeted therapeutics was further exacerbated by the lack of GSRCC-specific clinical trial data evaluating therapeutic agents within the broader category of gastric cancers. To date, a major challenge in the development of GSRCC-targeted ADC drugs is the identification of suitable membrane targets that effectively distinguish between malignant GSRCC cells and normal tissues. Our results demonstrated that PVR is a novel and promising ADC target for GSRCC due to its high tumoral overexpression and efficient cell internalization. PVR, also known as the poliovirus receptor (CD155), is a multifunctional protein involved in tumor progression and metastasis. Previous studies have shown that PVR is upregulated in various cancer types, including tumor-infiltrating myeloid cells, and its overexpression is associated with poor patient outcomes [16,29]. PVR’s role in the TME includes its ability to suppress anti-tumor immunity by engaging inhibitory receptors, further supporting its potential as an immunotherapy target [17,30].

Our presented study demonstrated that PVR-DXd is a promising ADC candidate for GSRCC by exhibiting high tumor specificity and therapeutic efficacy in preclinical models. Specificity of efficacy was confirmed through experiments in non-PVR-expressing cell lines. Additionally, PVR has been reported to play a key role in modulating the TME, particularly through interactions with immune cells, which suggests that targeting the PVR axis may enhance the efficacy of combination immunotherapies [31]. We also describe a comprehensive strategy for ADC target identification and payload selection by leveraging multi-omics data. Large-scale datasets encompassing, proteomics and transcriptomics provide critical insights to guide the rational selection of optimal ADC targets and payloads for drug discovery. The encouraging preclinical data obtained with PVR-DXd in GSRCC paves the way for further exploration of its clinical applications. Meanwhile, the intrinsic functions of PVR in tumor proliferation, invasion, and metastasis remain elusive, while its extrinsic immunoregulatory functions within the TME involve interaction with immune checkpoint receptor TIGIT. This dual role highlights PVR as a promising target for both ADC and immunotherapy [15,16]. PVR’s role in the TME includes its ability to suppress anti-tumor immunity by engaging inhibitory receptors, further supporting its potential as an immunotherapy target. Long-term toxicity and drug resistance assessments will be listed as limitations of the study and future research directions to be clarified.

This study has the following limitations. First, we did not perform a comprehensive ADC analytical and pharmacokinetic characterization, including orthogonal assessment of DAR distribution, aggregation, site occupancy, impurity profiling, or detailed evaluation of total antibody, conjugated antibody, and free DXd in circulation, nor formal analyses of serum stability, deconjugation kinetics, or efficacy stratified by absolute PVR antigen density; therefore, although our data are consistent with a predominantly target-dependent intracellular delivery mechanism, we cannot fully exclude contributions from systemic DXd release or bystander effects. Second, we did not define a validated protein-level cutoff or companion diagnostic framework for patient selection, bridge antigen binding capacity (ABC) values to IHC H-scores or percent-positive thresholds, or quantify the prevalence of PVR-high tumors in bona fide GSRCC cohorts. Third, we did not comprehensively address spatial heterogeneity of PVR expression across disease compartments (e.g., primary versus peritoneal or ascites lesions) or implement interobserver-controlled pathology scoring. Finally, this proof-of-concept study was not designed as a dose-finding or safety-optimization investigation and therefore does not provide a clinical starting-dose rationale based on allometry or PK/PD modeling, DXd-specific safety-monitoring plans (e.g., for ILD and neutropenia), or systematic evaluation of resistance mechanisms, rational combination strategies (including TIGIT/PD-1 blockade), and associated biomarkers such as ctDNA, soluble PVR, or lysosomal/cathepsin markers. These elements are being incorporated into our ongoing translational program and will be reported in future work.

## 5. Conclusions

This study identifies PVR as a promising ADC target for GSRCC and provides preclinical evidence supporting the efficacy and safety of PVR-DXd. Leveraging multi-omics approaches, we demonstrated the potential of PVR-DXd to selectively target GSRCC tumors while minimizing systemic toxicity. These findings underscore the importance of data-driven target selection in ADC development and provide a strong foundation for future clinical investigations of PVR-DXd in the treatment of GSRCC.

## Data Availability

All data relevant to the study are included in the article.

## Supplementary Materials

The following supporting information can be downloaded at: https://www.mdpi.com/article/10.3390/cancers18020270/s1, Figure S1: SEC-HPLC analysis of PVR IgG4 subtype antibody purity; Figure S2: SDS-PAGE verification of protein molecular weight of PVR IgG4 isoform antibody; Figure S3: SPR Screening for Highly Specific PVR Antibodies.
