# Supplementary material for: Poliovirus Receptor as a Potential Target in Gastric Signet-Ring Cell Carcinoma for Antibody-Drug Conjugate Development
Source: Cancers (Basel). 2026 Jan 15;18(2):270. doi: 10.3390/cancers18020270 (PMC12838934; doi:10.3390/cancers18020270)
Supplement: Supplementary file 1 [file cancers-18-00270-s001.zip › cancers-4046085-supplementary.pdf]

Supplementary appendix

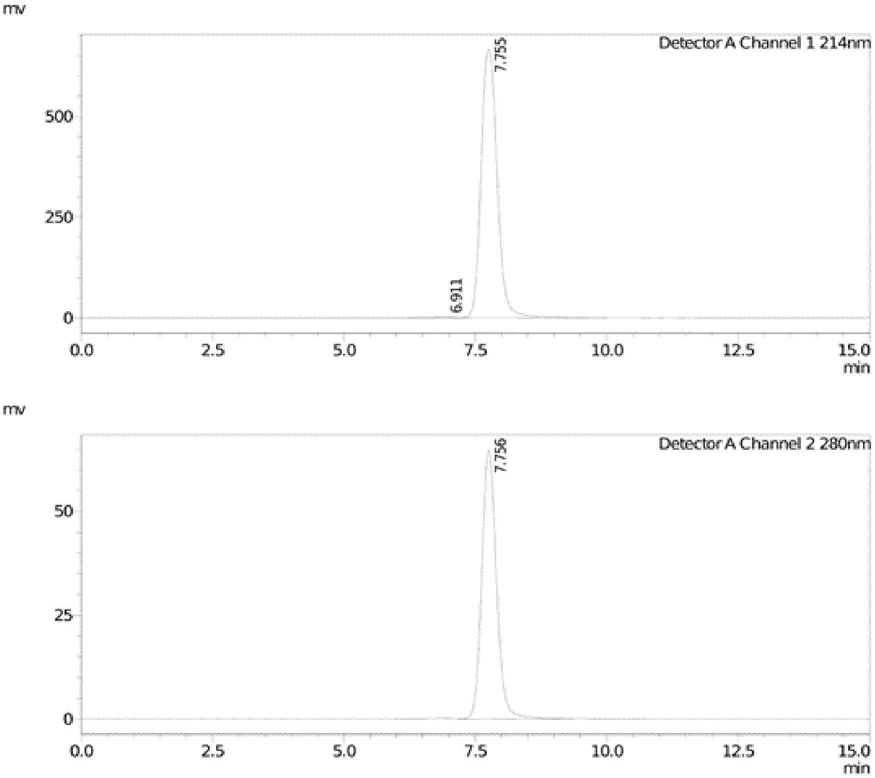

Figure S1 SEC-HPLC analysis of PVR IgG4 subtype antibody purity

Purity was analyzed by size exclusion high performance liquid chromatography (SEC-HPLC): 100% (214 nm detection) and 99.579% (280 nm detection). The retention time of the main peak was 7.755 min and the area share was 99.58% (214 nm), verifying no aggregation or degradation.

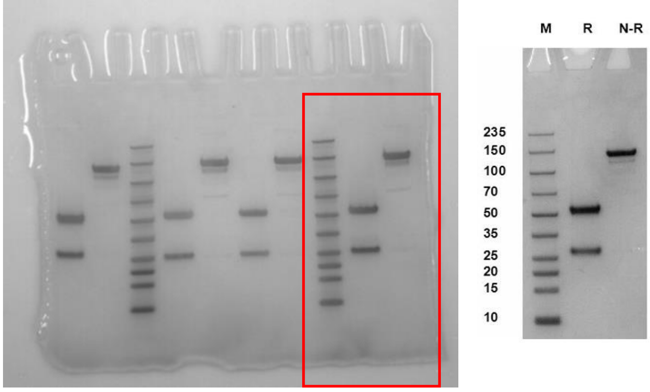

Figure S2 SDS-PAGE verification of protein molecular weight of PVR IgG4 isoform antibody

Protein molecular weight was verified by running sodium dodecyl sulfate polyacrylamide gel electrophoresis (SDS-PAGE). Under reducing conditions (SDS-PAGE): the antibody

depolymerized into heavy chain (49 kDa) and light chain (23 kDa),; non-reducing conditions (SDS-PAGE): the molecular weight of the intact antibody was 144 kDa, and SDS-PAGE electrophoresis showed a single band without contamination by heteroprotein.

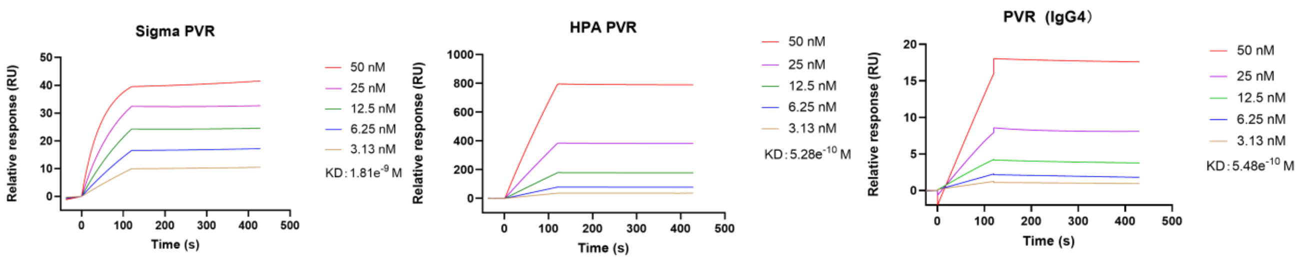

Figure S3 SPR Screening for Highly Specific PVR Antibodies

High-affinity PVR antibodies identified by surface plasmon resonance (SPR): Using SPR, we compared the binding performance of three PVR antibodies from Sigma (Cat#10109-H80H，Sino Biological), HPA (Cat#HPA064739), and an IgG4-subtype PVR antibody generated from the patented Baiying Bio sequence. The Sigma PVR antibody showed a KD of 1.81×10^{-9} M, whereas the HPA and the IgG4 PVR antibodies demonstrated markedly higher affinities, with KD values of 5.28×10^{-10} M and 5.48×10^{-10} M, respectively. Based on its high affinity and desirable IgG4 framework, the IgG4 PVR antibody was selected for subsequent studies.
